# Alcohol (70%) versus alcoholic chlorhexidine solution (0.5%) in skin antisepsis for neuraxial blocks: a randomized clinical trial

**DOI:** 10.1590/0100-6991e-20202633

**Published:** 2021-01-04

**Authors:** LUIZ CARLOS SOUZA TOSTES, ANA BEATRIZ ALKMIM TEIXEIRA LOYOLA, ADILSON DE OLIVEIRA FRAGA, LETÍCIA AZEVEDO GAZZI, LUIZ FRANCISLEY DE PAIVA, YARA JULIANO, DANIELA FRANCESCATO VEIGA

**Affiliations:** 1 - Universidade do Vale do Sapucaí (UNIVÁS), Mestrado Profissional em Ciências Aplicadas à Saúde - Pouso Alegre - MG - Brasil; 2 - Universidade do Vale do Sapucaí (UNIVÁS), Departamento de Farmácia - Pouso Alegre - MG - Brasil; 3 - Universidade do Vale do Sapucaí (UNIVÁS), Disciplina de Microbiologia - Pouso Alegre - MG - Brasil; 4 - Hospital e Maternidade Santa Paula, Programa de Residência Médica em Anestesiologia - Pouso Alegre - MG - Brasil; 5 - Universidade do Vale do Sapucaí (UNIVÁS), Faculdade de Medicina - Pouso Alegre - MG - Brasil; 6 - Universidade Santo Amaro (UNISA), Disciplina de Bioestatística - Santo Amaro - SP - Brasil; 7 - Universidade do Vale do Sapucaí (UNIVÁS), Disciplina de Bioestatística - Pouso Alegre - MG - Brasil; 8 - Universidade Federal de São Paulo (UNIFESP), Programa de Pós-graduação em Cirurgia Translacional - São Paulo - SP - Brasil

**Keywords:** Antisepsis, Chlorhexidine, Ethanol, Anesthesia Spinal, Anestesia Epidural, Antissepsia, Clorexidina, Álcool Etílico, Raquianestesia, Anestesia Epidural

## Abstract

**Objective:**

to compare the use of 0.5% alcoholic chlorhexidine and 70% alcohol in skin antisepsis for neuraxial blocks.

**Method:**

this is a non-inferiority randomized clinical trial, with two parallel arms. Seventy patients who were candidates for neuraxial block were randomly allocated to group A (n = 35), in whom antisepsis was performed with 0.5% alcoholic chlorhexidine, or to group B (n = 35), in whom we used 70% hydrated ethyl alcohol. Swabs were harvested for culture at three times: before antisepsis, two minutes after application of the antiseptic, and immediately after puncture. The samples were sown in three culture media and the number of colony forming units (CFU) per cm² was counted.

**Results:**

there was no difference between the groups regarding age, sex, body mass index, time to perform the block or type of block. There were no differences between groups in the CFU/cm² counts before antisepsis. There was less bacterial growth in group B two minutes after application of the antiseptic (p = 0.048), but there was no difference between the groups regarding the number of CFU/cm² at the end of the puncture.

**Conclusion:**

70% alcohol was more effective in reducing the number of CFU/cm² after two minutes, and there was no difference between the two groups regarding skin colonization at the end of the procedure. These results suggest that 70% alcohol may be an option for skin antisepsis before neuraxial blocks. **Trial registration:** ClinicalTrials.gov, NCT02833376.

## RESUMO

**Objetivo:**

comparar o uso de solução alcoólica de clorexidina 0,5% e de álcool 70% na antissepsia da pele para bloqueios do neuroeixo.

**Método:**

ensaio clínico randomizado de não inferioridade, com dois braços paralelos. Foram selecionados 70 pacientes candidatos à bloqueio do neuroeixo, randomicamente alocados para o grupo A (n=35), em que a antissepsia foi realizada com clorexidina alcoólica 0,5%, ou para o grupo B (n=35), em que se utilizou álcool etílico hidratado 70%. Foram coletadas, com swab, amostras para cultura em três momentos: antes da antissepsia, dois minutos após aplicação do antisséptico, e imediatamente após a punção. As amostras foram semeadas em três meios de cultura e foi contabilizado o número de unidades formadoras de colônias (UFC) por cm².

**Resultados:**

não houve diferença entre os grupos quanto à idade, ao sexo, ao índice de massa corporal, ao tempo para realização do bloqueio ou tipo de bloqueio. Também não houve diferenças entre os grupos na contagem de UFC/cm² antes da antissepsia. Constatou-se menor crescimento bacteriano no grupo B dois minutos após aplicação do antisséptico (p=0,048), mas não houve diferença entre os grupos quanto ao número de UFC/cm² ao final da punção.

**Conclusão:**

o álcool 70% mostrou-se mais efetivo em reduzir o número de UFC/cm² após dois minutos, e não houve diferença entre os dois grupos quanto à colonização da pele ao final do procedimento. Esses resultados sugerem que o álcool 70% pode ser opção para antissepsia da pele antes de bloqueios do neuroeixo. **Registro ensaio clínico**: ClinicalTrials.gov, NCT02833376.

## INTRODUCTION

Neuraxial blockades are among the most performed anesthetic procedures, but there are no official data on their numbers carried out in the world, and neither in Brazil. It is often the first choice of anesthesia for surgical procedures, spinal anesthesia being used mainly for surgery in the lower limbs, perineum and abdomen^1^.Epidural anesthesia can also be used, with the difference that the blockade can be achieved by segments, such as only the trunk or abdomen. This type of anesthetic procedure has the main advantage of maintaining the patient’s spontaneous ventilation and awareness^1^.

 As it is an invasive procedure, to prevent bacterial contamination, antisepsis measures are necessary both on the skin at the puncture site and on the anesthesiologist’s hands, in addition to barrier methods, such as the use of sterile gloves, cap and mask^2^ ^,^ ^3^.

The human microbiota varies in different places in the human body, displaying a higher concentration of bacteria in some sites, and this can influence the action of antiseptics on the bacterial microbiota^4^. The lumbar region has one of the lowest concentrations of bacterial colonies when compared with other areas of the human body^4^.

Antisepsis is the process of destroying the vegetative form of microorganisms (pathogenic or not) present in living tissues. It is a set of measures used to destroy or inhibit the growth of microorganisms existing in the superficial (transient microbiota) and deep (resident microbiota) layers of the skin and mucous membranes. Such measures involve the application of germicidal agents, the antiseptics^5^. Antiseptics should have immediate antimicrobial action, persistent residual effect, and should not be toxic, allergenic, or irritating. Different antiseptics are used in clinical practice, such as 70% alcohol, chlorhexidine alcoholic solution, polyvinylpyrrolidone (PVP), and iodine alcohol^6^.

The antiseptic activity of ethyl alcohol occurs by denaturation of proteins and removal of lipids, including the envelopes of some viruses. To achieve maximum germicidal activity, the alcohol must be diluted with water, which allows protein denaturation. The recommended concentration to achieve greater germ-killing speed is 70%^6^.

Chlorhexidine is a germicide from the group of biguanides, which is more effective at pH between five and eight, and acts better against gram-positive bacteria than gram-negative ones or fungi. It has immediate action and residual effect, in addition to low potential for toxicity and photosensitivity to contact, being poorly absorbed by intact skin. Its mechanism of action involves increasing the permeability of the cell wall, causing precipitation of intracellular components. This action is potentiated by alcohol, so the alcoholic solution is more effective^7^.

 Infections of the neuroaxis after anesthesia are rare, but serious. These complications are generally cited as case reports. The exact incidence is unknown, but they can result in devastating morbidity and mortality, including abscess formation, meningitis, or spinal cord compression secondary to abscess formation^8^ ^-^ ^12^. The Brazilian Society of Anesthesia recommends the use of alcoholic chlorhexidine solution for skin antisepsis for neuraxial blockades^2^. In 2014, the United Kingdom Anesthetists Associations released security guidelines for skin antisepsis for neuraxial anesthesia, which do not recommend the use of a specific antiseptic. The suggestion is to use a fast-acting antiseptic, with minimal side effects, and to take care not to contaminate the needle with the used antiseptic and wait for the antiseptic to dry on the skin, thus avoiding complications of the introduction of the antiseptic in the neuraxis^13^ ^,^ ^14^. 

Although the guidelines include a vast literature review, no specific studies have been described comparing antiseptics for skin antisepsis in neuraxial blockades^13^. In fact, no scientific evidence has been found to support the use of a particular antiseptic for this purpose. Thus, the objective of this clinical trial was to compare the effect, on skin colonization, of 70% hydrated ethyl alcohol and 0.5% alcoholic chlorhexidine solution used for skin antisepsis in neuraxial blockades.

## METHODS

This is a randomized clinical trial of non-inferiority, in a single center, with two parallel branches unbeknownst to the microbiologist. The study project was approved by the Ethics in Research Committee of the Universidade do Vale do Sapucai - UNIVÁS (CAAE: 54214116.3.0000.5102), and fully complied with the Helsink declaration. The study was registered on the ClinicalTrials.gov platform, under number NCT02833376.

To calculate the sample size, we used data from the study by Sato et al. (1996)^15^. We applied the one-tailed Student t test. With significance level of 5% and 90% power, the calculated number of patients per group was 35.

 We selected patients who would already undergo surgical procedures at the Hospital e Maternidade Santa Paula, in Pouso Alegre - MG. We included patients of both sexes, aged between 18 and 65 years, who had an indication of neuraxial anesthesia (spinal or epidural) by the attending anesthesiologist. We did not include patients with a body mass index (BMI) above 35 kg/m^2^, with a diagnosis of infection in any part of the body, who had made use of antibiotics in the prior seven days, and who had skin lesions at the puncture site. Patients who met the eligibility criteria were informed about the study during a pre-anesthetic visit and were invited to participate. Only those who accepted by signing the free and informed consent form were included. 

We randomly allocated patients to group A (n = 35), in which antisepsis was performed with 0.5% chlorhexidine alcoholic solution, or to group B (n = 35), in which we used 70% hydrated ethyl alcohol for skin antisepsis. For the allocation of patients to the groups, was generated a random sequence with the BioEstat 5.0 software (Mamirauá Institute, Brazil), and the allocation concealment was guaranteed by opaque and sealed numbered envelopes, opened in the operating room immediately before puncture antisepsis.

After hand antisepsis with 2% chlorhexidine and using sterile gloves, the anesthesiologist poured 30 mL of the respective antiseptic in a sterile vat, from a sealed and individualized cannolo. The antiseptic was applied to the skin, from the puncture site extending to an area with a radius of 20 cm. This procedure was repeated twice, and the puncture was performed two minutes after the second application.

The collection of samples for microbiological evaluation was performed in the operating room, with a sterile swab applied in a standardized way over an area of 5x5 cm, delimited by a fenestrated sterile drape placed on the puncture site, in three moments: before antisepsis, two minutes after the application of the antiseptic, and immediately after the blockade. Each swab was placed in a sterile tube containing 1 mL of saline. The samples were kept refrigerated, taken to the laboratory, and processed within a maximum of four hours.

We used standardized microbiological methods^16^. The swab soaked in saline containing the collected material was stirred, and 0.1 mL of this solution extracted, which was later inoculated with a loop on three plates containing the media: Plate Counter Agar (PCA) for growth of Gram-positive and Gram-negative bacteria; Mannitol Agar, selective for Gram-positive germs; and Teague Agar, to isolate Gram-negative microorganisms. The plates were incubated in an aerobic environment at 37° C. After 48 hours, a microbiologist who was unaware of groups’ allocation read the number of colony-forming units (CFU).

### Statistical analysis

Given the nature of the variables studied and the variability of the values found, we used non-parametric tests^17^. For the analysis, we used the BioEstat 5.3 software (Instituto Mamirauá, Pará and Amazonas, Brazil), and the level of rejection of the null hypothesis was fixed at 5% (α? 0.05).

We used the Mann-Whitney test to compare the two independent groups (A and B) regarding age, BMI and puncture time. We applied the Chi-square test to compare the two groups regarding distribution by sex and type of anesthesia (spinal or epidural).

We also used the Mann-Whitney test to compare groups A and B for skin colonization at each time (before antisepsis, after two minutes, and immediately after puncture). For intra-group evaluation, we used the Friedman Analysis of Variance by Ranks to compare the number of CFU in the three collection moments (before antisepsis, after two minutes, and immediately after the puncture). Whenever there was statistical significance, we applied the multiple comparisons test to determine which differed significantly from the others. We performed such analyzes independently for each culture medium.

## RESULTS

**Figure 1:**
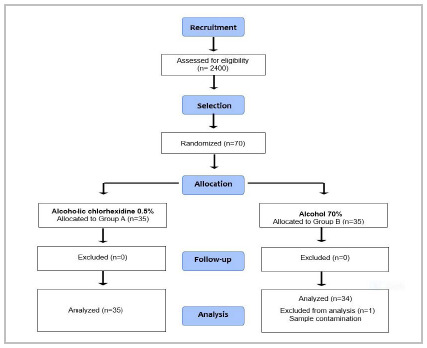
CONSORT diagram of patient flow in the study ^18^ .

We included 70 patients in the study. Figure 1 shows the flow of patients in the study. There was no exclusion. We excluded the result of cultures of one patient from Group B after puncture due to contamination of the samples.

Both groups were homogeneous in terms of the main demographic characteristics. The puncture median time was three minutes for both groups, also with no statistical difference, and the most frequent type of anesthesia was spinal, in over 90% of patients in both groups (Table 1).

**Table 1:** Main demographic and blockade characteristics, in both groups.

Variables	Group A	Group B	p-value
Age (years)			0.518*
Range	19 - 63	22 - 64	
Median	41	44	
BMI (kg/m2)			0.837*
Range	22 - 31.4	20.3 - 32.6	
Median	27.5	27.3	
Sex [n (%)]			1.000**
Female	12 (34)	12 (34)	
Male	23 (66)	23 (66)	
Puncture time (minutes)			0.672*
Range	1 - 22	1 - 20	
Median	3	3	
Blockade type [n (%)]			0.643**
Spinal anesthesia	33 (94)	32 (92)	
Epidural anesthesia	2 (6)	3 (8)	

BMI - body mass index; * Mann-Whitney test; ** Chi-square test.

Tables 2 to 4 show the results regarding skin colonization in groups A and B, as well as the intra-group statistical comparison (before antisepsis, after two minutes, and immediately after puncture), for the media PCA, Mannitol Agar (Gram +), and Teague Agar (Gram -), respectively.

**Table 2:** Number of CFU in the PCA medium in groups A and B at the three moments (Friedman Analysis of Variance by Ranks), and comparison between groups at each moment (Mann-Whitney test).

PCA			
CFU number			
	Group A	Group B	A x B
	n=35	n=35	(Mann-Whitney)
Pre-antisepsis			
Range	0 - 3000	0 - 2560	p = 0.565
Median	30	35	
After 2 minutes			
Range	0 - 60	0 - 60	p = 0.048
Median	0	0	
After puncture			
Range	0 - 150	0 - 580	p = 0.322
Median	0	0	
Pre x 2min x After	p= 0.003	p < 0.000	
(Friedman)	Pre > 2min and final	Pre > 2min and final	

*PCA - Plate Counter Agar; CFU - Colony-forming units.*

**Table 3:** Number of CFU in the Mannitol Agar medium in groups A and B in the three moments (Friedman Analysis of Variance by Ranks), and comparison between groups in each moment (Mann-Whitney test).

Mannitol Agar (Gram +)			
CFU number			
	Group A	Group B	A x B
	n=35	n=35	(Mann-Whitney)
Pre-antisepsis			
Range	0 - 3000	0 - 2450	p = 0.719
Median	10	20	
After 2 minutes			
Range	0 - 10	0 - 10	p = 0.418
Median	0	0	
After puncture			
Range	0 - 100	0 - 960	p = 0.710
Median	0	0	
Pre x 2min x After	p = 0.0003	p < 0.0001	
(Friedman)	Pre > 2min and final	Pre > 2min and final	

*CFU - Colony-forming units.*

**Table 4:** Number of CFU in the Teague Agar medium in groups A and B in the three moments (Friedman Analysis of Variance by Ranks), and comparison between groups in each moment (Mann-Whitney test).

Teague Agar (Gram )			
CFU number			
	Group A	Group B	A x B
			(Mann-Whitney)
Pre-antisepsis			
Range	0 - 950	0 - 0	p = 0.683
Median	0	0	
After 2 minutes			
Range	0 - 0	0	-
Median	0	0	
After puncture			
Range	0	0	-
Median	0	0	
Pre x 2min x After	p = 0.918	-	
(Friedman)			

*CFU - Colony-forming units.*

## DISCUSSION

Evidence-based medicine consists of using the scientific method to obtain evidence to guide health care decisions. Expert reports are not as reliable as the results of well-conducted studies, which in turn are also inferior to the results of a set of well-conducted studies. Thus, levels of evidence should be classified according to strength, or level of reliability. Stronger evidence will have more weight in clinical decision-making^19^.

Although the literature is not specific regarding the occurrence of infectious complications resulting from neuraxial anesthesia, there are intrinsic factors, such as hematogenic transmission^20^, as well as extrinsic ones. Among the extrinsic factors, we highlight the bacterial migration through the needle puncture path, contaminated syringes, catheters, injection of local anesthetics, or failed antiseptic techniques. The migration of bacteria through the needle path is the main source of infection in neuraxial blockades^14^ ^,^ ^21^ ^-^ ^23^.

The neuraxial anesthesia is an invasive procedure, hence requiring specific care to prevent contamination and antisepsis measures, both of the skin, puncture site, and the anesthesiologist’s hands, and the use of barrier methods, such as sterile gloves, cap, and mask^24^. In the present study, all professionals were using appropriate barrier methods.

The lack of adequate skin preparation when performing invasive procedures promotes infection. Despite widespread knowledge of the importance of antisepsis before performing a neuraxial blockades, there is still no consensus on the most appropriate technique or the ideal antiseptic solution^3^ ^,^ ^25^.

The lumbar region has a density of microorganisms per cm^2^ lower than other parts of the body. This fact is related to a smaller number of sebaceous glands; in this region, there is a predominance of aerobic Gram-positive bacteria^4^. The results of the present study corroborate this, since there was evidence of greater growth in media selective for Gram-positive microorganisms.

There are bacteria that are located deep in the skin, in places where antiseptics often do not penetrate, due to lipophilic secretions in the stratum corneum. For this reason, the use of alcohol-based antiseptics is always indicated, due to the degreasing action, which provides greater penetration capacity and efficiency in the eradication of deeper bacteria^23^ ^,^ ^26^ ^,^ ^27^. Alcohol-based antiseptics have rapid action, denaturing proteins and removing lipids, with the ability to penetrate the stratum corneum, follicles and orifices of the sebaceous glands, sites where there is a higher concentration of bacteria^6^ ^,^ ^23^ ^,^ ^28^ ^,^ ^29^.

Studies have shown that one must wait a minimum of two minutes to antiseptic action after the application^8^ ^,^ ^23^ ^,^ ^30^. The protocol of the present study took into account this minimum time of two minutes between antisepsis and puncture, which proved to be sufficient to reduce the skin microbiota. The puncture time varied between one and 22 minutes, with a median of three, and even after the longest punctures, there was no significant bacterial growth, demonstrating that the two studied antiseptics showed satisfactory residual action for the procedure in question, which is fast.

Other authors have also demonstrated the satisfactory residual action of these two antiseptics in the short term, comparing the effect of alcoholic chlorhexidine and 70% alcohol. They collected samples with swabs of intact skin from different regions of the body, 10 minutes, six hours and 24 hours after application, and did not observe statistical difference in colonization after 10 minutes or six hours. However, they found that after 24 hours, chlorhexidine maintained the residual effect, which did not happen with alcohol, with statistical significance^28^.

Alcohol at 70% was more effective in reducing the number of CFU/cm^2^ after two minutes, and there was no difference between the two groups regarding skin colonization at the end of the procedure. These results suggest that 70% alcohol may be an option for skin antisepsis before neuraxial anesthesia.

The limitations of the present study include its performance in a single center, with a small sample size. When calculating the sample size, we found no studies comparing the use of alcohol and chlorhexidine in skin antisepsis for neuraxial blockades. Thus, the calculation was based on the proportion of positive cultures observed in the study by Sato et al. (1996)^15^, who compared 0.5% chlorhexidine alcoholic solution with 10% povidone-iodine for skin antisepsis in lumbar surgery (5.7% and 32.4% of positive cultures, respectively). A multicenter study, with a larger number of patients, and eventually the design of a pragmatic clinical trial, could provide stronger evidence to support the clinical practice of antisepsis for neuraxial anesthesia. 
